# Circular RNAs as molecular bridges: dual regulation of ferroptosis and immunity in cancer

**DOI:** 10.3389/fimmu.2026.1833421

**Published:** 2026-06-15

**Authors:** Songbai Xu, Peiyi Liang, Tie Lin, Guangxin Zhang, Xiying Fu, Yicun Wang

**Affiliations:** 1 Department of Neurosurgery, First Hospital of Jilin University, Changchun, China; 2 Department of Medical Research Center, Second Hospital of Jilin University, Changchun, China; 3 Department of Cerebral Surgery, The First Affiliated Hospital of Harbin Medical University, Harbin, China; 4 Department of Thoracic Surgery, Second Hospital of Jilin University, Changchun, China; 5 Department of Endocrinology, Second Hospital of Jilin University, Changchun, China

**Keywords:** cancer therapy, circular RNA, ferroptosis, ferroptosis-immunity crosstalk, molecular bridge, tumor immune microenvironment

## Abstract

Circular RNAs (circRNAs) are covalently closed single-stranded RNA molecules generated by back-splicing events of precursor mRNAs, characterized by superior structural stability, strict tissue specificity, and diverse regulatory functions. Ferroptosis is an iron-dependent, lipid peroxidation-driven regulated cell death (RCD) distinct from classical apoptosis and necrosis; remodeling of the tumor immune microenvironment (TIME) directly modulates cancer progression and therapeutic efficacy. Both are core targets in cancer biology and translational oncology. Emerging evidence shows circRNAs act as “molecular bridges” to simultaneously regulate ferroptosis and anti-tumor immunity via miRNA sponging, protein interaction/scaffolding, and *de novo* encoding of functional peptides. This review systematically elaborates the molecular mechanisms of circRNA-mediated ferroptosis-immunity crosstalk in cancer, where ferroptosis and anti-tumor immunity reciprocally regulate each other through inflammatory signals and immune effectors. CircRNAs act as “molecular bridges” by simultaneously targeting key nodes in both pathways via a single regulatory event, a concept defined here as “dual regulation”. we focused on three major regulatory modalities, and explores their potential as non-invasive diagnostic biomarkers and novel therapeutic targets, providing new perspectives for precision cancer therapy via dual targeting of ferroptosis and immune pathways.

## 1 Introduction

Cancer progression is driven by the dysregulated coordination of multiple genes and pathways, with aberrant cell death programs and immune evasion as two interconnected core hallmarks promoting tumorigenesis, metastasis, and therapy resistance (1). CircRNAs, a unique class of non-coding RNAs (ncRNAs), have become a tumor biology research hotspot due to their covalently closed loop structure [lacking 5’-caps and 3’-poly(A) tails] that confers high exonuclease resistance and stable expression in tumor cells (2, 3). They extensively participate in oncogenic pathway activation, tumor suppressor inactivation, metabolic reprogramming, and metastatic cascades. They are aberrantly expressed in lung, hepatocellular, breast and other solid tumors, modulating tumor initiation, progression and therapy resistance via miRNA sponging, protein interactions and functional peptide encoding (4). Ferroptosis is implicated in regulating cancer development, metastasis and chemo/radio/immunotherapy resistance, while the TIME forms an immunosuppressive phenotype via regulatory T (Treg) cell infiltration, myeloid-derived suppressor cell (MDSC) accumulation and immune checkpoint upregulation, enabling immune escape and limiting immunotherapy efficacy (5). Notably, recent studies reveal circRNAs act as “molecular bridges” linking these two seemingly independent processes, exerting dual regulatory effects on ferroptosis-immunity crosstalk via multiple mechanisms (6–9). This review focuses on circRNA-mediated crosstalk among ferroptosis, immunity and inflammation in the TIME, highlights the molecular mechanisms and therapeutic implications, and discusses the significance of developing precision cancer diagnostic and therapeutic strategies.

## 2 Structural features and regulatory mechanisms of circular RNAs

CircRNAs are generated from pre-mRNAs through back-splicing events, forming covalently closed loops without traditional 5’ caps and 3’ polyadenylate tails (10). Their unique conformation confers robust resistance to exonuclease-mediated degradation, ensuring prolonged intracellular stability (11). CircRNA biogenesis is tightly regulated by intronic complementary sequences that facilitate intramolecular base pairing and RNA-binding proteins (RBPs) that promote back-splicing (5, 12, 13). CircRNAs display distinct tissue-, cell- and developmental stage-specific expression patterns, with aberrant expression documented in multiple malignancies (10, 14). They regulate tumor progression via four core mechanisms (10): (i) miRNA sponging (ceRNA mechanism); (ii) protein interaction and scaffolding; (iii) transcriptional/post-transcriptional regulation; and (iv) *de novo* encoding of functional peptides. These diverse mechanisms enable circRNAs to modulate tumor cell proliferation, apoptosis, metabolism and metastasis. Moreover, their high stability and strict specificity make them promising tumor biomarkers and therapeutic targets, and in-depth mechanistic research provides a theoretical basis for developing circRNA-based precision diagnostic tools and therapies (15).

## 3 Bidirectional crosstalk between ferroptosis and immunity in cancer

Ferroptosis and anti-tumor immunity are closely intertwined in the TIME, with their dynamic interaction profoundly affecting cancer progression and therapeutic response (16). On the one hand, ferroptotic tumor cells release damage-associated molecular patterns (DAMPs) (e.g., ATP, HMGB1, ROS), activating innate and adaptive anti-tumor immunity via ferroptosis-induced immunogenic cell death (ICD) (17). On the other hand, IFN-γ secreted by activated CD8^+^ T cells suppresses the expression of key ferroptosis regulators such as SLC7A11 and GPX4, enhancing tumor cell ferroptosis susceptibility and synergizing with immune cytotoxicity (8). Inflammation acts as a critical bridge connecting ferroptosis and immunity: ferroptotic cells activate the NLRP3 inflammasome via DAMPs, inducing secretion of pro-inflammatory cytokines IL-1β and IL-18. These cytokines shape the TIME by recruiting myeloid immune cells and modulating T cell activation/effector functions, forming a dynamic “ferroptosis-immunity-inflammation” regulatory axis that circRNAs coordinately modulate at multiple levels (18). We define “dual regulation of ferroptosis and immunity” as a single circRNA’s ability, through one molecular mechanism to concurrently affect both ferroptosis sensitivity and immune cell function.

## 4 Circular RNAs as molecular bridges: dual regulation of ferroptosis and immunity

**Figure 1:**
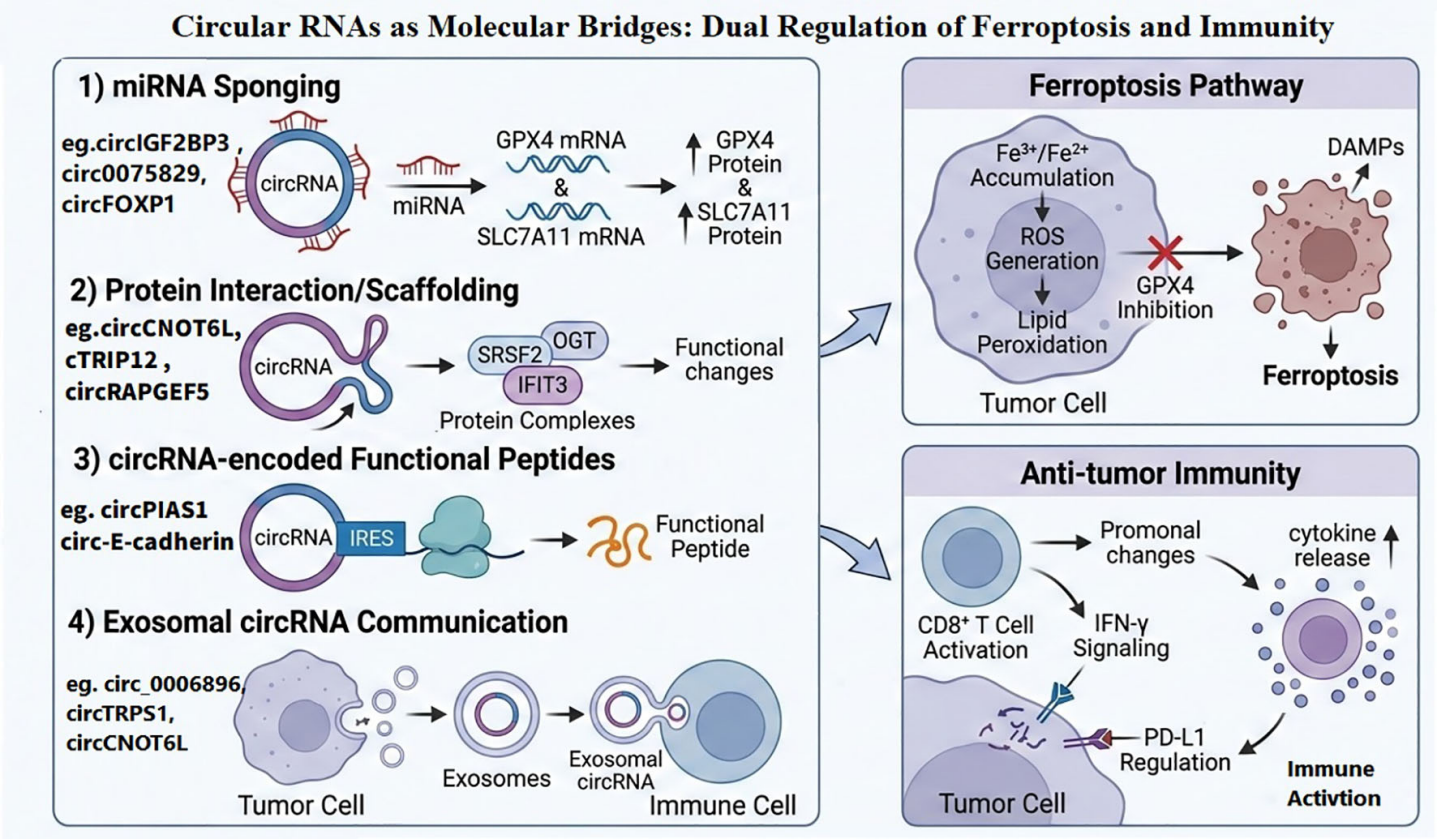
Schematic overview of circRNAs acting as molecular bridges that coordinately regulate ferroptosis and immunity. Four major mechanisms are illustrated: (1) miRNA sponging – circRNAs (e.g., circIGF2BP3) sequester miRNAs to derepress SLC7A11 and GPX4, thereby regulating ferroptosis and immunity; (2) protein scaffolding – circRNAs (e.g., circCNOT6L) recruit protein complexes (e.g., SRSF2-IFIT3) to modulate downstream effectors such as OGT and IGF1R, regulate ferroptosis and immunity; (3) circRNA-encoded functional peptides (e.g., circPIAS1) promote CD8^+^ T cell activation and IFN-γ signaling, influencing PD-L1 regulation, while inhibiting ferroptosis; (4) exosomal circRNAs (e.g., circ_0006896) mediate intercellular communication between immune cells and tumor cells. The ferroptosis pathway is also depicted.

CircRNAs serve as core molecular bridges integrating ferroptosis and immune regulation via three predominant mechanisms: miRNA sponging, protein interaction/scaffolding, and functional peptide encoding. In each mechanism, the circRNA acts on a single molecular event that concurrently affects both a ferroptosis-related target (e.g., GPX4) and an immunity-related target (e.g., PD-L1). Additionally, exosomal circRNAs play a critical role in intercellular communication and remote regulation of these two processes (**Figure 1**; **Table 1**).

**Table 1:** Representative circRNAs as molecular bridges in ferroptosis-immunity dual regulation.

Cancer type	circRNA	Mechanism	Ferroptosis effect	Immunity effect	Inflammation	Ref
Prostate cancer	circCNOT6L	Sponges miR-143-5p → SRSF2/SLC7A11	Inhibition	Unknown	Unknown	(21)
Colon cancer	circ_0075829	Sponges miR-330-5p → TCF4	Inhibition (↑GPX4, xCT)	Immune escape (↑PD-L1)	Un known	(19)
Gallbladder cancer	circFOXP1	Sponges miR-4477a → PD-L1	Inhibition	Immune escape	Ginsenoside Rg3	(20)
AML	exo-circ_0006896	Binds HDAC1	Inhibition	Suppresses immunity	HDAC1	(28)
Pancreatic cancer	cTRIP12	Binds OGT/PERK	Inhibition (↑FTH)	Immune escape (↑PD-L1)	PERK-ER stress	(22)
Lung adeno	circ_BBS9	Binds IFIT3	Promotion (↑ROS)	Immune activation	IFIT3-IFN	(23)
Melanoma	circPIAS1-108aa	STAT1 SUMOylation	Inhibits immunogenic	Immune escape	STAT1	(26)
Breast cancer	C-E-cad (peptide)	Activates EGFR→CXCL8	Unknown	MDSC recruitment	CXCL8	(27)
Endometrial cancer	circRAPGEF5	Binds RBFOX2→TFRC	Inhibition (↓labile Fe)	Unknown	Unknown	(24)
Colorectal cancer	circRAPGEF5	Encodes neoepitope → activates CD8^+^ T cells	Unknown	Immune activation	Unknown	(39)

↑ indicates upregulation or increase, while ↓ indicates downregulation or decrease.

### 4.1 miRNA sponging-mediated regulation

CircRNAs act as competing endogenous RNAs (ceRNAs) to sequester shared miRNAs, thereby simultaneously derepressing mRNAs involved in ferroptosis and immune pathways. Unlike protein interaction or peptide encoding, miRNA sponging relies on sequence complementarity and is typically cytoplasmic. For example, in colorectal cancer (CRC), circ_0075829 targets miR-330-5p to upregulate TCF4, simultaneously inhibiting ferroptosis (via GPX4/xCT upregulation) and increasing PD-L1, impairing CD8^+^ T cell function and promoting immune escape (19). In gallbladder cancer, circFOXP1 sponges miR-4477a to upregulate PD-L1 and suppress ferroptosis, and Ginsenoside Rg3 targets this axis to activate CD8^+^ T cells and induce ferroptosis (20). These findings indicated that a single circRNA, via one miRNA sponge event, can coordinately regulate two distinct groups of target genes.

### 4.2 Protein interaction-mediated regulation

Here, circRNAs directly bind to specific RNA-binding proteins (RBPs) or other protein partners via defined structural domains, functioning as scaffolds or decoys to modulate protein function. This mechanism does not involve miRNA competition; instead, it alters protein activity, localization, or alternative splicing. In prostate cancer, circCNOT6L binds SRSF2 to modulate SLC7A11 alternative splicing, conferring ferroptosis resistance (21). In pancreatic cancer, cTRIP12 interacts with OGT and PERK to increase FTH/PD-L1 O-GlcNAcylation, simultaneously inhibiting ferroptosis and promoting immunosuppression (22). In lung adenocarcinoma, circ_BBS9 binds IFIT3 to promote ferroptosis (via ROS elevation) and enhance CD8^+^ T cell infiltration (23). In endometrial cancer, circRAPGEF5 associates with RBFOX2 to regulate TFRC alternative splicing, reducing the labile iron pool (LIP) to inhibit ferroptosis (24). These studies highlight circRNAs as versatile protein interaction scaffolds, regulating ferroptosis/immunity-related proteins via distinct domains and revealing how tumor cells coordinate stress responses to adapt to the hostile TIME. In prostate cancer, circCCDC719–13 inhibits HSP90 to induce TAM ferroptosis and M2-to-M1 reprogramming, suppressing tumor progression. This expands circRNA-protein scaffolds to HSP90 in the immune microenvironment (25).

### 4.3 Peptide-mediated regulation

A subset of circRNAs contain internal ribosome entry sites (IRES) and open reading frames (ORFs), enabling cap-independent translation into functional peptides that independently modulate ferroptosis and immunity. This mechanism is distinct from the previous two because the circRNA itself is not the direct effector; rather, its encoded peptide carries out the dual regulation. In melanoma, circPIAS1 encodes circPIAS1-108aa, which enhances STAT1 SUMOylation and blocks its phosphorylation, relieving SLC7A11/GPX4 suppression (inhibiting immunogenic ferroptosis) and suppressing CD8^+^ T cell activation (26). In breast cancer, circ-E-cadherin encodes C-E-cad, which activates EGFR signaling to promote CXCL8 secretion and MDSC recruitment, while inhibiting ferroptosis (27). Peptide-mediated regulation offers high specificity and can be targeted without affecting the parent circRNA.

### 4.4 Exosomal circRNAs and remote regulation

While circRNAs can act within a single cell, exosomal circRNAs extend dual regulation across different cell types in the TIME. Exosomes encapsulate circRNAs and deliver them to recipient cells, enabling remote paracrine signaling. And the dual regulation can occur not only cell-autonomously but also non-cell-autonomously. In acute myeloid leukemia (AML), leukemic cell-derived exosomal circ_0006896 is taken up by CD8^+^ T cells, binding HDAC1 to reduce IFN-γ secretion, while conferring ferroptosis resistance to leukemic blasts (28). In bladder cancer, exosomal circTRPS1 modulates glutamine metabolism/ROS balance via the miR-141-3p/GLS1 axis, inducing CD8^+^ T cell exhaustion and altering tumor ferroptosis sensitivity to achieve metabolic-immune dual suppression (29). Exosomal circRNAs also link inflammation with ferroptosis/immunity by modulating NF-κB/STAT3 signaling in recipient cells. The circRNA-ferroptosis-immunity axis involves complex feedback loops (e.g., CD8^+^ T cell-derived IFN-γ upregulates circPIAS1, forming a negative feedback loop that suppresses T cell function and immunogenic ferroptosis (26)) and spatiotemporal specificity: nuclear circRNAs (e.g., circCNOT6L) regulate pre-mRNA splicing (21),while cytoplasmic circRNAs act as miRNA sponges/scaffolds (30); the same circRNA exerts distinct effects in tumor vs. immune cells; circCNOT6L is upregulated in metastatic vs. primary prostate cancer (21); and hypoxia/inflammatory cytokines dynamically modulate circRNA expression/function (31).

## 5 Clinical translational potential

### 5.1 Diagnostic and prognostic biomarkers

CircRNAs’ high stability, tissue specificity and biofluid detectability make them ideal non-invasive liquid biopsy biomarkers. In melanoma, circPIAS1 expression correlates with immune checkpoint blockade (ICB) resistance (26); in NSCLC, exosomal circUSP7 predicts anti-PD-1/PD-L1 efficacy (32); in AML, exosomal circ_0006896 indicates poor immunotherapy response (28); in CRC, circ_0075829 correlates with ferroptosis resistance/immune escape and serves as a prognostic marker (19). Dual regulatory circRNAs and their exosomal forms thus enable non-invasive monitoring of immunotherapy response and ferroptosis-related resistance in cancer patients. However, current studies are mostly retrospective. Prospective validation and standardized detection protocols for circRNAs in biofluids are lacking. Future multi-center prospective cohorts are needed to establish clinical utility and regulatory pathways.

### 5.2 Therapeutic targets

Targeting dual regulatory circRNAs is a promising strategy to overcome therapy resistance. Three preclinically validated approaches include: (1) Direct circRNA targeting: ASOs against circPIAS1 restore immunogenic ferroptosis and enhance PD-1 inhibitor efficacy in melanoma (26); circCNOT6L siRNA combined with Erastin inhibits prostate cancer organoid viability (21). (2) Combination with existing therapies: cTRIP12 knockdown plus ferroptosis inducers/CTLA-4 inhibitors suppresses pancreatic cancer growth (22); circMGA upregulation synergizes with PD-1 inhibitors to enhance bladder cancer immunotherapy response (33). (3) Peptide targeting: Inhibitors of circPIAS1-108aa/C-E-cad enhance ICB efficacy without affecting parent circRNA functions (26, 27). These multi-level strategies provide new avenues to overcome ferroptosis resistance and immune escape. So far, no circRNA therapy for ferroptosis-immunity dual regulation has entered clinical trials.

### 5.3 Clinical translation challenges

Despite great potential, several challenges limit clinical application: (1) Specificity and delivery: CircRNAs share high homology with linear transcripts, making specific targeting difficult (34); efficient, tissue-specific delivery systems remain a bottleneck (35). (2) Context-dependent functions: CircRNAs exert distinct effects in different cell types/tumor stages, requiring patient molecular stratification (36). (3) Safety: Exogenous nucleic acid drugs may trigger immunogenic reactions, and long-term circRNA modulation may perturb normal cell function, necessitating systematic preclinical safety evaluation (37). (4) Limited clinical validation: Most findings are based on preclinical models, lacking large-scale, prospective multi-center trials to verify clinical value (38). Overcoming these challenges requires interdisciplinary collaboration across RNA biology, nanomedicine, tumor immunology and translational research.

## 6 Conclusion and perspectives

CircRNAs are critical molecular bridges integrating ferroptosis-immunity crosstalk in cancer, coordinately regulating these processes via miRNA sponging, protein scaffolding, peptide encoding, exosomal communication and feedback loops, with aberrant expression profoundly affecting tumor progression and therapy response. Inflammation as a third regulatory axis adds complexity and new therapeutic opportunities. Key dual regulatory circRNAs (e.g., circCNOT6L, circ_0075829) and their encoded peptides (e.g., circPIAS1-108aa, C-E-cad) deepen our understanding of cancer biology and open novel precision therapy avenues. Future research should focus on three key directions: (1) Elucidating global circRNA regulatory networks of ferroptosis-immunity crosstalk via high-throughput sequencing and multi-omics; (2) Developing efficient, safe tissue-specific delivery systems based on nanotechnology and synthetic biology; (3) Conducting large-scale prospective multi-center clinical trials to validate circRNAs’ diagnostic/prognostic value and therapeutic efficacy. With advances in RNA biology and translational oncology, circRNA-based precision therapy targeting the ferroptosis-immunity axis will become a promising novel strategy for cancer treatment.
